# Evaluation of entropy features and classifier performance in person authentication using resting-state EEG

**DOI:** 10.3389/fnins.2025.1651501

**Published:** 2025-11-04

**Authors:** Renyu Yang, Ling zhang, Yuanmei Peng, Boming Zhong, Lixing Hou, Jinhui Peng, Baoguo Xu, Renhuan Yang

**Affiliations:** ^1^School of Big Data and Artificial Intelligence, Guangdong University of Finance & Economics, Guangzhou, China; ^2^Key Laboratory of Collaborative Innovation in Digital Economy, Guangdong University of Finance & Economics, Guangzhou, China; ^3^Guangzhou Vocational College of Technology & Business, Guangzhou, China; ^4^Red Cross Hospital of Yulin City, Yulin, China; ^5^Yulin Orthopedic Hospital of Integrated Traditional Chinese and Western Medicine, Yulin, China; ^6^School of Instrument Science and Engineering, Southeast University, Nanjing, China; ^7^College of Information Science and Technology, Jinan University, Guangzhou, China

**Keywords:** bioinformation technique, electroencephalogram (EEG), entropies, person authentication, classifier

## Abstract

**Introduction:**

Resting-state electroencephalogram (EEG) presents a promising biometric modality due to its inherent liveness detection and resistance to spoofing, addressing critical vulnerabilities in conventional systems. However, its deployment faces fundamental trade-offs among accuracy, robustness, and hardware efficiency, particularly concerning optimal electrode configuration, discriminative feature extraction, and classifier generalization.

**Methods:**

To address these challenges, this study systematically evaluates thirteen entropy measures—including spectral entropy (SpEn), refined composite multiscale entropy, fuzzy entropy, and sample entropy (SaEn) etc.—alongside six classifiers (Quadratic Discriminant Analysis (QDA), Random Forests and Support Vector Machines etc.) for person authentication. Using 32-channel EEG recordings from 26 healthy participants under rigorous leave-one-out cross-validation (LOOCV), we quantified the impact of electrode selection and feature-classifier pairing.

**Results:**

Key findings demonstrate: QDA classifier achieved peak performance of 96.8% accuracy using 30 electrodes. Critically, a streamlined 9-electrode portable configuration retained 96.1% accuracy, demonstrating robust performance with reduced hardware requirements. SpEn measure exhibited superior biometric discriminability compared with other entropy measures, exceeding SaEn by 13.8 percentage points.

**Conclusion:**

These results advance the design of portable EEG biometric devices while highlighting entropy features’ scalability.

## 1 Introduction

Person authentication, verifying an identity claim through 1:1 biometric matching against stored templates, faces growing demands for enhanced security, privacy, and convenience. Conventional biometrics [e.g., fingerprint (Crouse et al., 2015), hand geometry (Bera et al., 2015), iris textures (Kumar and Passi, 2010)] and voice patterns (Pal and Saha, 2015) exhibit well-documented vulnerabilities including spoof susceptibility, template aging, and postmortem misuse risks. In contrast, electroencephalogram (EEG) signals offer inherent advantages as a biometric modality: spoofing resistance due to the non-replicable nature of neural dynamics, automatic postmortem invalidation, and continuous liveness monitoring capabilities (Billeci et al., 2013; Unnisa et al., 2025). These advantages, position EEG as a compelling biometric modality for next-generation security systems.

However, the transition of EEG-based authentication from laboratory environments to real-world deployment faces significant practical constraints (Unnisa et al., 2025). First, environmental fragility plagues resting-state EEG protocols—the dominant approach for its operational convenience (Di et al., 2019; Wang et al., 2016). Although passive acquisition of neural signatures avoids cognitive burdens (Kim and Kim, 2019; Waili et al., 2019), these protocols exhibit higher susceptibility to motion artifacts and ambient noise than stimulus-driven paradigms, critically compromising signal-to-noise ratios (SNR) in uncontrolled environments. For instance, Suppiah et al. (2016) demonstrated that even basic state transitions (eyes open/closed) introduce significant intra-subject variability in single-channel systems.

Second, hardware-accuracy tradeoffs impose scalability constraints (Carrión-Ojeda et al., 2021). Electrode count directly governs spatial resolution and system complexity: while high-density electrode arrays (64 + channels) achieve excellent discriminative performance, they incur prohibitive costs and operational complexity that hinder scalability (Barayeu et al., 2020). Conversely, consumer-grade devices (typically ≤16 channels) sacrifice accuracy for practical usability (Zhang et al., 2023). Necessitating application-specific optimization between performance and practicality (Kawala-Janik et al., 2015; Wu and Zhao, 2023).

The third challenge involves feature selection and classifier construction. Feature extraction challenges arise from EEG’s inherent nonlinearity and non-stationarity. Feature extraction from EEG signals can be performed in the temporal, spatial, and spectral domains, each offering distinct insights into the underlying neural activity (Ramzan and Dawn, 2023). In the time domain, autoregressive (AR) models are commonly used to extract features, while power spectral density (PSD) (Wang et al., 2019) and wavelet-based methods are employed in the frequency domain. Despite the utility of these methods, many traditional approaches may not fully capture the rich information in EEG signals. Entropy measures address this gap by quantifying signal uncertainty and complexity, offering robust analytical frameworks for EEG (Tenev et al., 2025; Yang et al., 2025). Recent research has explored advanced feature extraction techniques, such as entropy-based methods and time-frequency analysis, to improve the accuracy and robustness of EEG-based biometric systems (Hernández-Álvarez et al., 2022; Min et al., 2017; Vella et al., 2017). Multivariate Multiscale Entropy (MvMSE) captures the complexity of EEG signals across both temporal scales and spatial electrodes, enabling the characterization of spatiotemporal complexity profiles in multivariate signals (Lewandowska et al., 2023). Multivariate dispersion entropy (MvDPE) can simultaneously capture temporal dynamics and inter-channel interactions, providing stable estimates even for short time series (Azami et al., 2019). Through various variants including wavelet entropy, Shannon entropy, generalized Escort-Tsallis entropy, and Rényi entropy, these entropy measures effectively quantify signal complexity, providing new perspectives for deeply understanding and utilizing the individual characteristics of EEG signals.

In EEG-based authentication, classification methods primarily include machine learning (ML) and deep learning (DL) approaches. Traditional machine learning techniques, such as support vector machines (SVM), random forests (RF), and K-nearest neighbors (KNN), are widely used due to their simplicity, interpretability, and computational efficiency. For instance, Kaewwit et al. (2017) integrated AR modeling, independent component analysis, and KNN classifiers reaching 92.7% accuracy under 10-fold cross-validation. Hernández-Álvarez et al. (2022) achieved 91.1% accuracy using a RF approach. DL models, including convolutional neural networks (CNN), recurrent neural networks (RNN), long short-term memory (LSTM), and gated recurrent units (GRU), have emerged as a powerful tool for enhancing classification accuracy. For instance, the combination of CNN and LSTM models, as well as bidirectional LSTM with neural networks (BLSTM-NN), has demonstrated superior performance, with accuracies reaching 97.6%. Despite these advantages, DL models face challenges including high computational demands, substantial data requirements, and limited interpretability compared to ML alternatives.

Despite growing interest in EEG-based biometrics, the non-stationarity and non-linearity of EEG signals complicate feature extraction and classification, limiting practical deployment. Although prior studies improve accuracy, key gaps persist—including suboptimal feature selection, classifier robustness, and performance degradation with reduced electrodes. To address this, we systematically evaluate entropy features and classifiers for resting-state EEG person authentication, aiming to balance practicality and high performance. Key contributions are: (1) Entropy Feature Evaluation: Thirteen entropy measures (e.g., approximate entropy (ApEn), refined composite multiscale entropy (RCMSE), fuzzy entropy (FuEn) and spectral entropy (SpEn) etc.) are assessed, revealing SpEn ‘s superior efficacy for short, noisy EEG data in distinguishing individuals. (2) Classifier Benchmarking: Six algorithms are tested, with QDA and ensemble methods showing consistent robustness under resting-state conditions. (3) Optimized Configuration: Using only nine electrodes and QDA, we achieve 96.1% accuracy (26-subject dataset, leave-one-out validation), enhancing reliability while minimizing hardware complexity.

The remainder of this paper is organized as follows. Section 1 introduces the background, motivation, and research objectives. Section 2 details the methodology, including data acquisition, data preprocessing, entropy-based feature extraction, and classification algorithms. Section 3 presents the experimental results and performance analysis. Section 4 discusses the findings in the context of existing literature and highlights future research directions. Finally, Section 5 concludes the study and summarizes the key contributions.

## 2 Materials and methods

**Figure 1:**
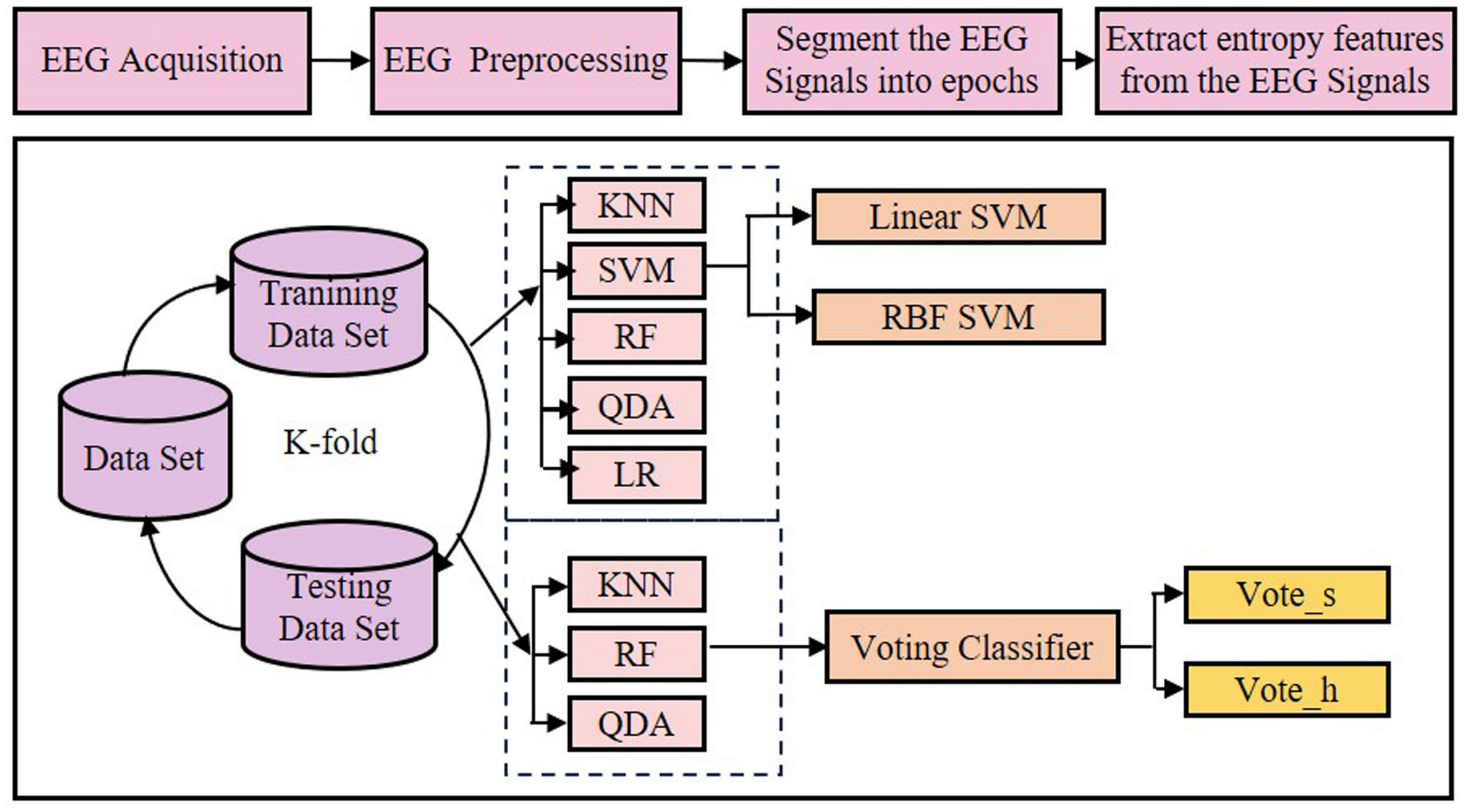
Workflow of the proposed study and Channel location.

The experimental workflow follows five sequential phases: (1) EEG data acquisition, (2) preprocessing of raw recordings including artifact removal, (3) segmentation into fixed-length epochs, (4) development of a classification model using entropy-derived features (e.g., SaEn), and (5) performance evaluation of classifiers. This pipeline is schematically detailed in Figure 1. Each authentication trial constitutes a binary classification task: given a claimed identity, the system verifies whether a test EEG epoch matches the enrolled template for that individual (genuine user) or not (imposter).

### 2.1 Data acquisition

Twenty-six healthy adults (aged 18 to 24 years; 12 males and 14 females) with no history of neurological disorders substance abuse history, or sleep abnormalities were recruited. The experiment was approved by the Academic Ethics Committee in accordance with the Declaration of Helsinki. Written informed consent was obtained from all participants. Based on the experimental design requirements for EEG biometric authentication, participants were seated in a sound-attenuated, electromagnetically shielded room while maintaining an awake, eyes-open state for 10 min to capture stable neural dynamics while minimizing fatigue-induced artifacts and standardizing data comparability with established resting-state EEG protocols (Polich, 2007), after which only the final 5 min were analyzed to ensure participants reached a stabilized resting baseline post-initial adaptation and exclude early-stage artifacts including electrode settling and transient alertness fluctuations (Shamlo et al., 2015). EEG data were acquired using a 32-channel Ag/AgCl electrode cap (Brain Products GmbH) positioned according to the 10–20 international system. The linked mastoids (A1/A2) served as reference electrodes to minimize frontal bias, enhance posterior signal fidelity, and reduce volume-conduction artifacts in temporal regions (Hu, 2017; Mu et al., 2017; Shamlo et al., 2015). Signals were digitized at 1000 Hz with 24-bit resolution, while electrode impedances were maintained below 5 kΩ throughout all recordings.

### 2.2 Data preprocessing

All EEG data processing was performed sequentially in MATLAB R2020a using EEGLAB toolbox and custom scripts. (1) Data first underwent 0.15 Hz high-pass filtering to eliminate baseline drift (2) Ocular artifacts were then corrected via Independent Component Analysis (ICA) using the extended Infomax algorithm, with components showing strong correlation ( ∣r∣ > 0.7) with EOG channels being rejected. (3) Muscle artifacts were reduced through wavelet denoising (Daubechies-4 wavelet, 8–40 Hz range) using adaptive soft thresholding. (4) Powerline noise at 50 Hz was removed with a 4th-order Butterworth notch filter (2-Hz bandwidth). Continuous data from the final 5 min were segmented into non-overlapping 1-s epochs—a duration chosen to balance entropy feature stability in the θ -band (requiring ≥3 cycles) and sensitivity to microstate dynamics (typically 80–120 ms per state). This pipeline yielded a final dataset of 7,800 artifact-free epochs (300 epochs per subject across 26 subjects), processed within the Neuroscan Scan 4.3 environment. The experiments were conducted on a computer equipped with an Intel Core i7-7700K CPU @ 4.20GHz, 16GB of memory, and an NVIDIA GeForce GTX 1080Ti GPU.

### 2.3 Feature extraction

Thirteen entropy features—ApEn, RCMSE, FuEn, kolmogorov entropy (KE), multiscale entropy (MSE), MvDPE, permutation entropy (PE), sample entropy (SaEn), SpEn, symbolic transfer entropy (STE), wavelet log energy entropy (WLE) and wavelet packet energy entropy (WPE), MvMSE,—were extracted from each 1-s EEG epoch, grouped into thirteen complementary paradigms addressing distinct aspects of EEG non-stationarity, with parameters rigorously optimized for electrophysiological relevance:

1. Time-Domain Regularity Metrics (ApEn, SaEn, FuEn): Quantify local signal predictability through instantaneous amplitude variations. Parameters of ApEn/SaEn were set m = 2, r = 0.2 × SD (SD: epoch-level standard deviation) based on established EEG benchmarks (Yang al et al., 2025). This configuration optimally captures microstate transitions (80–120 ms duration) while ensuring cross-subject comparability (Hu and Min, 2018). SaEn improves upon ApEn by eliminating self-matches for enhanced consistency in short time-series (Richman and Moorman, 2000), while FuEn employs Gaussian fuzzy membership $D_{\mathrm{ij}}=\exp(-x_{i}-x_{j}/r)$ for noise robustness (Xiang et al., 2015).
2. Spectral/Time-Frequency Disorder Metrics (SpEn, WLE, WPE): Map oscillatory instability through energy distribution irregularities. SpEn applies Shannon entropy to normalized FFT power spectra (256-point Hamming window, 50% overlap) (Kannathal et al., 2005). WLE and WPE use 5-level Daubechies-4 wavelet decomposition, computing log-energy entropy ( $-\sum_{j}E_{j}\log E_{j}$ ) and full-tree energy distribution, respectively.
3. Ordinal-Pattern Complexity Metrics (PE, MvDPE): Detect macro-scale temporal reorganizations via rank-order dynamics. PE parameters (m = 5, *τ* = 4, s = 2) capture θ−γ cross-frequency coupling (Min et al., 2017). MvDPE extends this with multivariate symbolic analysis (c = 6 symbol classes, d = 3 delay) for spatial pattern characterization (Azami et al., 2019).
4. Multi-Scale & Information Dynamics (MSE, RCMSE, MvMSE, STE, KE): Characterize cross-hierarchy dynamics. Multi-scale entropies (MSE/RCMSE) apply scale factor*τ* = 20 to SaEn-based coarse-grained sequences, covering δ−γ band dynamics while RCMSE reduces variance through composite averaging (Wu et al., 2014). MvMSE adopts m = 2, r = 0.2 × SD (consistent with time-domain metrics) for multichannel analysis. STE quantifies directional information flow via first-difference binarization (Δx > 0 → ‘1’) (Vicente et al., 2011). KE estimates system randomness with m = 6 optimized through grid search (Schouten et al., 1994).

The parameterization of 13 Entropy metrics for EEG-based person authentication are shows as Table 1. Parameters without neurophysiological baselines were optimized via grid search (e.g., m∈ (4.8) for KE), maximizing identifiability index I across 26 subjects using 10-fold cross-validation. Physiological adaptations (*m* = 2, τ = 20, *r* = 0.2 × SD) and noise mitigation (FuEn/STE/RCMSE) ensured robust biometric authentication.

**Table 1:** Parametric specifications of entropy metrics.

Entropy type	Key parameters	Physiological basis
ApEn/SaEn/FuEn	*m* = 2, *r* = 0.2 × SD	Microstate dynamics (80–120 ms)
PE	*m = 5, τ = 4, s = 2*	θ−γ cross-frequency coupling
MvDPE	*c* = 6, *d* = 3	Non-Gaussian spatial patterns
MSE/RCMSE	τ = 20, SaEn base	δ−γ band multiscale dynamics
MMSE	*m* = 2, *r* = 0.2 × SD, *τ* = 20	Cross-channel & cross-scale interactions
STE	Δ*x*>0 → ‘1’	Directed information flow
SpEn	None	Shannon entropy on normalized FFT spectrum
WLE/WPE	Daubechies-3, Levels = 5	Wavelet subband/full-tree energy entropy
KE	m = 6	Grid search

### 2.4 Classification model

Given the absence of a standardized EEG classification framework, we rigorously evaluated six distinct classifiers to ensure robustness across diverse subjects and entropy features: KNN, Logistic Regression (LR), Support Vector Machines (SVM), Random Forests (RF), Quadratic Discriminant Analysis (QDA), and an ensemble voting classifier. This multi-algorithm approach mitigates model-specific biases and leverages complementary strengths for person authentication. As a non-parametric instance-based method, KNN avoids assumptions about data distribution, making it suitable for EEG’s non-stationary characteristics. It implements learning based on the K-nearest neighbors of the training samples, with K empirically determined to be 7 in our study. LR was selected for its efficiency in high-dimensional spaces and probabilistic class outputs. Regularization strength (inverse of penalty weight), optimizing generalization via grid search. SVM, belonging to the class of generalized linear classifiers, relies heavily on kernel functions. When training an SVM classifier with the Radial Basis Function (RBF) kernel, particular emphasis must be placed on two crucial parameters: c and g. A smaller value of c typically leads to a smoother decision surface, enhancing generalization, whereas a larger c aims to classify all training examples more accurately, albeit potentially at the expense of overfitting. The parameter g reflects the influence weight of individual training examples, playing a vital role in shaping the decision boundary. Based on an exhaustive grid search method (Lameski et al., 2015), we determined optimal values of c = $2^{7}$ and g = $2^{-3}$ for our study. RF, an ensemble of decision trees, ensure diversity by allowing each tree to depend on independently sampled random vector values, thereby maintaining a uniform distribution across all trees in the forest. In our study, we meticulously selected the number of trees to be 300 and the number of input variables considered for each split to be 2, based on comprehensive validation. QDA seeks an optimal linear combination of features that statistically maximizes the separation between objects belonging to different classes, characterized by a quadratic decision boundary. This technique is particularly robust, leveraging posterior distributions to accurately estimate the class of a given test sample. Lastly, the voting classifier integrates diverse classifiers through fusion methods, enhancing the overall robustness and accuracy of the classification system. Specifically, soft fusion (Vote_s) employs the average predicted probabilities to determine the target subject, offering a probabilistic interpretation of the classification results. Conversely, hard fusion (Vote_h) utilizes a majority voting approach, providing a deterministic outcome based on the collective decision of the ensemble classifiers. By leveraging these fusion methods, we further strengthen the reliability and performance of our classification framework.

### 2.5 Evaluation method

To assess the performance of the proposed classifier, detection quality was evaluated using three complementary metrics: (1) leave-one-out cross-validation (LOOCV) was applied at the subject level to prevent data leakage, ensuring robustness in feature set identification; (2) average accuracy, precision, recall, F1-score, and confusion matrix were calculated across combinations of feature sets, classifiers, channels, and subject groups to quantify the overall detection efficacy;(3) receiver operating characteristic (ROC) analysis was adopted as the primary metric for binary classification tasks. As defined by ROC theory: ROC curves plot sensitivity (true positive rate) against 1-specificity (false positive rate) under varying classification thresholds. The optimal threshold corresponds to the point closest to the top-left corner of the curve. The area under the ROC curve (AUC) provides a quantitative performance measure, where higher values indicate superior separability (AUC = 1: perfect classifier; AUC < 0.5: worse than random). ROC curves were generated per subject by iteratively adjusting the decision threshold for binary predictions. AUC values were computed using the trapezoidal rule.

## 3 Results

**Figure 2:**
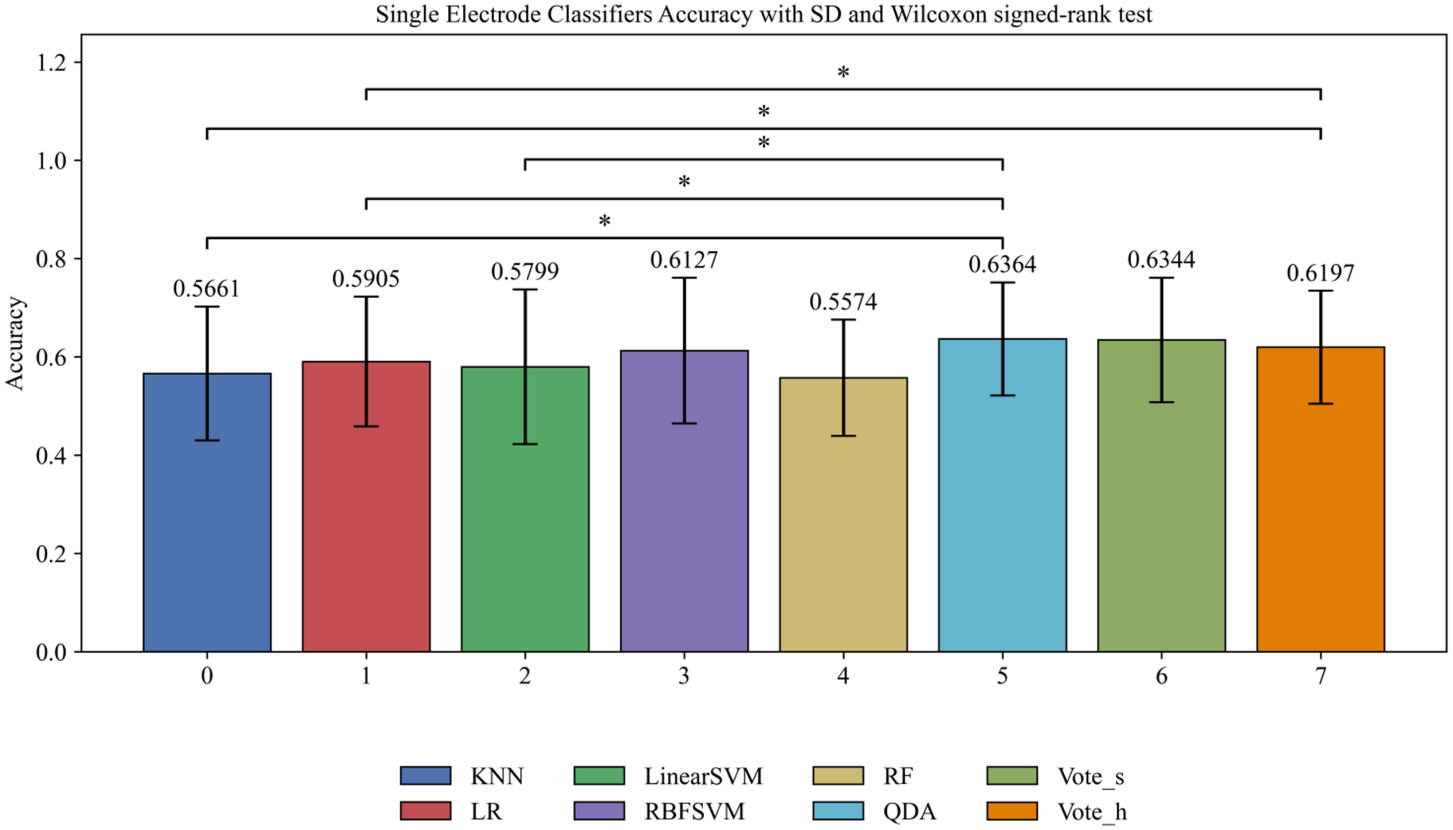
Comparison of average identification accuracy among different classifiers using a single electrode combined with all thirteen entropy feature sets, wilcoxon signed-rank test (*p* < 0.001).

To evaluate classifier performance in person authentication, we compared the average accuracy of across six classifiers utilizing EEG signals from a single electrode combined with thirteen entropy-based feature sets. Figure 2 demonstrates that classifier selection critically influenced performance. QDA achieved peak accuracy (63.6%), while RF performed poorest (55.7%). The results demonstrated that the mean accuracy using single electrode remained suboptimal for practical deployment, even with multi-feature fusion. Prior to inferential analysis, we assessed the normality of paired differences and homogeneity of variances. While the assumption of equal variance was met, the normality assumption was violated for most comparisons. Consequently, the nonparametric Wilcoxon signed-rank test was employed for paired comparisons (Candia-Rivera and Valenza, 2022). To mitigate Type I error inflation due to multiple testing, false discovery rate (FDR) correction was applied to all *p*-values. Wilcoxon signed-rank test (*n* = 26 subjects) revealed several significant differences after FDR adjustment: QDA surpassed KNN (*W* = 0.000, *p* < 0.001, $r_{\mathrm{rb}}$ = 1.000, Median_diff = −0.070), LR (*W* = 1.000, *p* < 0.001, $r_{\mathrm{rb}}$ = 0.994, Median_diff = 0.034), and Linear SVM (*W* = 3.000, *p* < 0.001, $r_{\mathrm{rb}}$ = 0.983, Median_diff = 0.041). Similarly, the Vote_h outperformed KNN (*W* = 0.000, *p* < 0.001, $r_{\mathrm{rb}}$ = 1.000, Median_diff = 0.063) and LR (*W* = 0.000, *p* < 0.001, $r_{\mathrm{rb}}$ = 1.000, Median_diff = 0.049). No significant differences were observed between. KNN and Linear SVM (*W* = 12.000, *p* = 0.037, $r_{\mathrm{rb}}$ = − 0.675, Median_diff = −0.044); QDA and Vote_h (*W* = 138.000, *p* = 0.353, $r_{\mathrm{rb}}$ = 0.2137, Median_diff = 0.003). Overall classifier ranking by performance was: QDA ≈ Vote_s > Vote_h > RBFSVM > LR > Linear SVM > KNN > RF.

**Figure 3:**
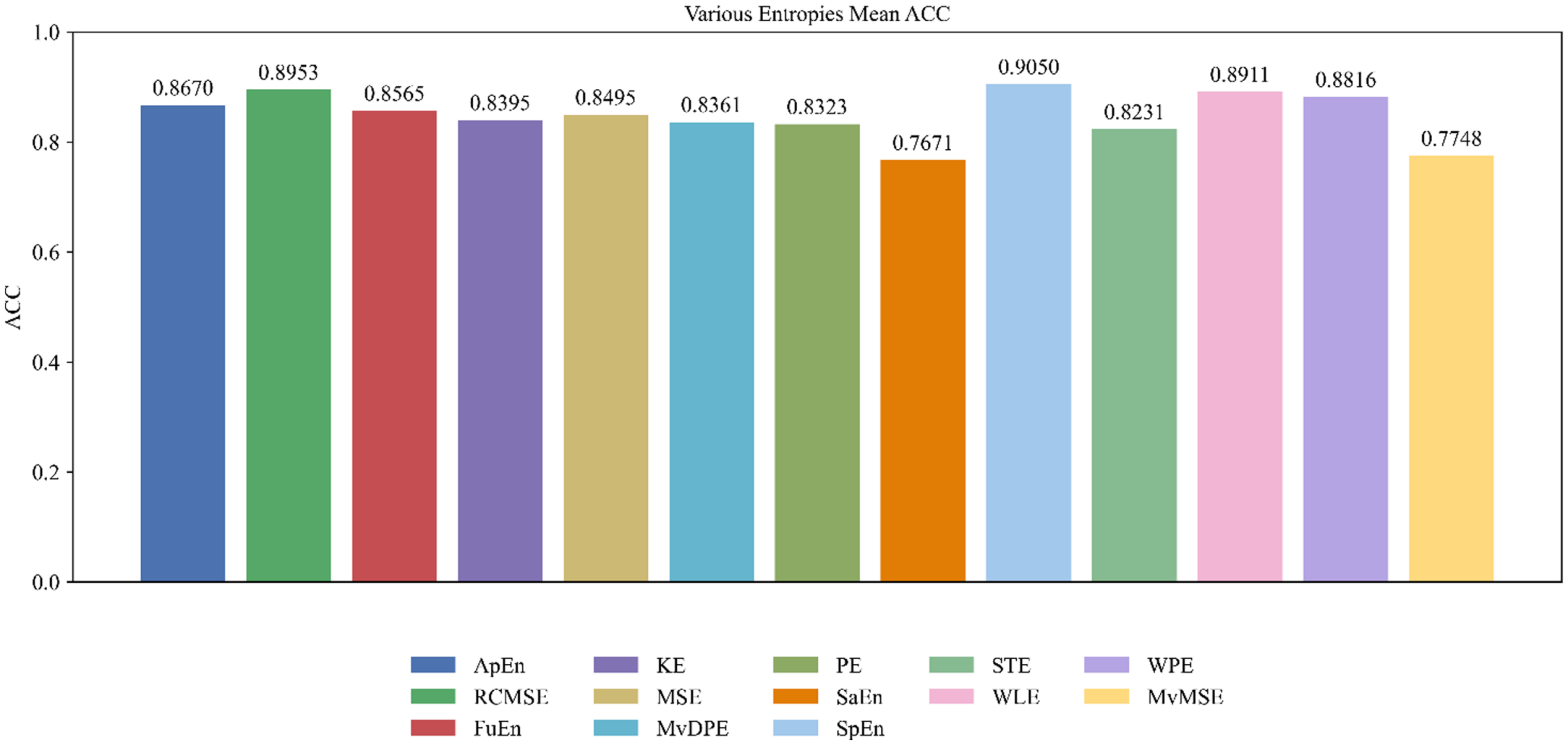
Mean classification accuracy per feature type across eight classifiers using resting-state EEG signals from all 30 electrodes.

To evaluate the impact of various entropy features on person authentication performance, we computed the average classification accuracy across eight classifiers for each feature type, utilizing resting-state EEG signals from all 30 electrodes. As shown in Figure 3, feature type markedly affected authentication accuracy. The SpEn feature achieved the highest biometric authentication accuracy (90.5%), followed by RCMSE (89.5%) and WLE at 89.1%. WPE and ApEn also exhibited robust performance, with accuracies of 88.2 and 86.7%, respectively. Moderate results were observed for FuEn (85.6%), MSE (84.9%) and KE (84.0%). Conversely, MvDPE (83.6%), PE (83.2%), STE (82.3%), MvMSE (77.5%), and SaEn (76.7%) exhibited comparatively lower classification performance. Among thirteen entropy features, SpEn demonstrated superior performance, exceeding SaEn by 13.8 percentage points in classification accuracy, thereby establishing SpEn as the optimal feature for characterizing individual uniqueness in resting-state EEG data.

**Figure 4:**
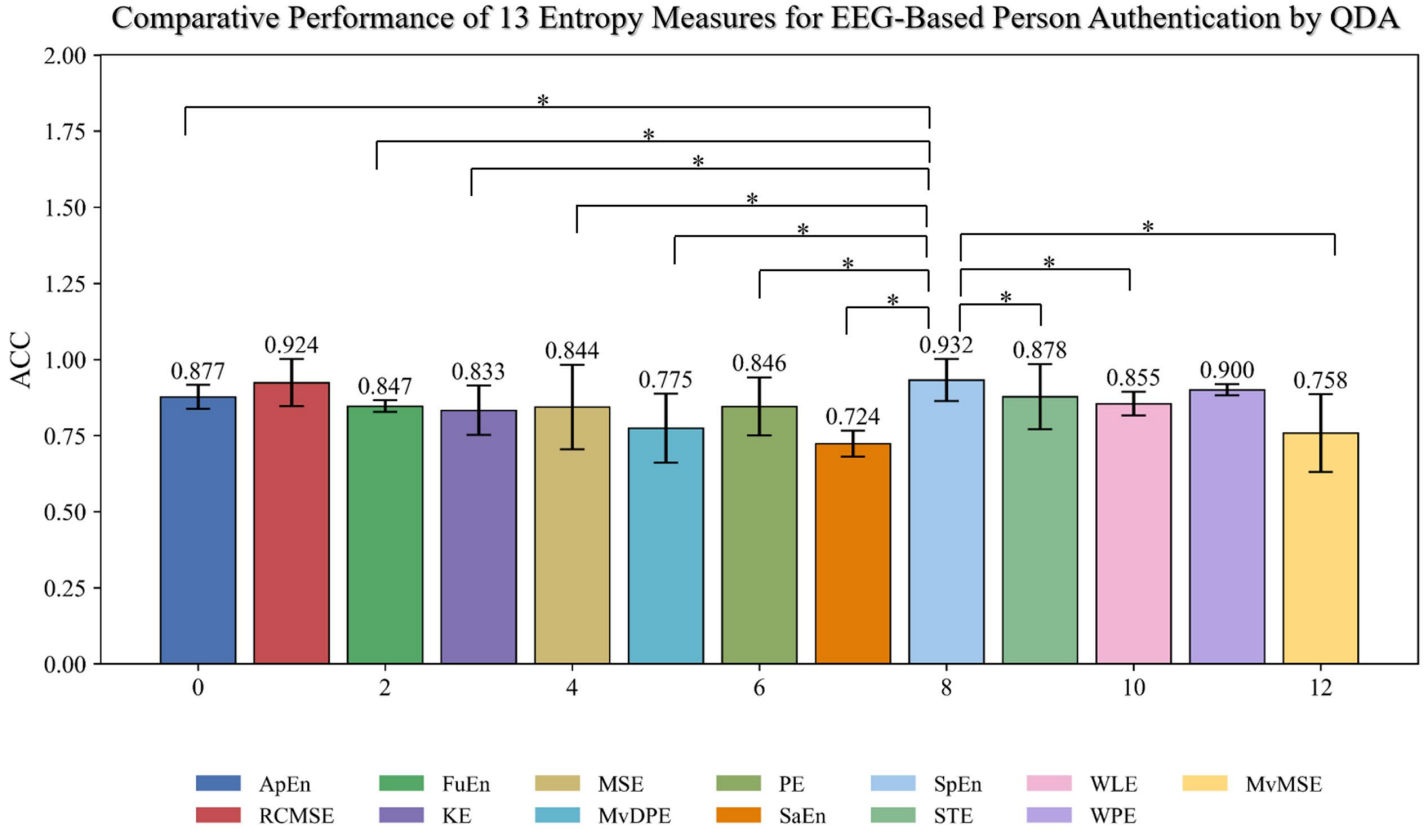
Comparison of the performance (accuracy with standard deviation) of all 13 entropy features within the QDA framework revealed significant performance differences, as confirmed wilcoxon signed-rank test (*p* < 0.001).

Furthermore, we integrated the classifier selected from prior analysis (QDA) with 13 distinct entropy measures to evaluate their person authentication performance using resting-state EEG. As depicted in Figure 4, the classification accuracies achieved were: SpEn (93.2%), RCMSE (92.2%), WPE (90.0%), STE (87.8%), ApEn (87.7%), WLE (85.5%), FuEn (84.7%), PE (84.6%), MSE (84.4%), KE (83.3%), MvDPE (77.5%), MvMSE (75.8%), and SaEn (72.4%). Statistical validation, following normality and homogeneity of variance tests which indicated non-normal distributions, was performed using the wilcoxon signed-rank test (*n* = 26 subjects). Results confirmed that entropy selection critically governs classification accuracy (*p* < 0.001). SpEn significantly outperforming all other entropies except RCMSE and WPE (*p* < 0.001 for all other comparisons), with the most pronounced disparity observed between SpEn and SaEn (*W* = 0.000, *p* < 0.001, $r_{\mathrm{rb}}$ = 1.000), indicating an exceptional effect size. These results align with the trends in Figure 3 but demonstrate marginal improvement due to QDA optimization, confirming the robustness of entropy features across classifier configurations.

**Figure 5:**
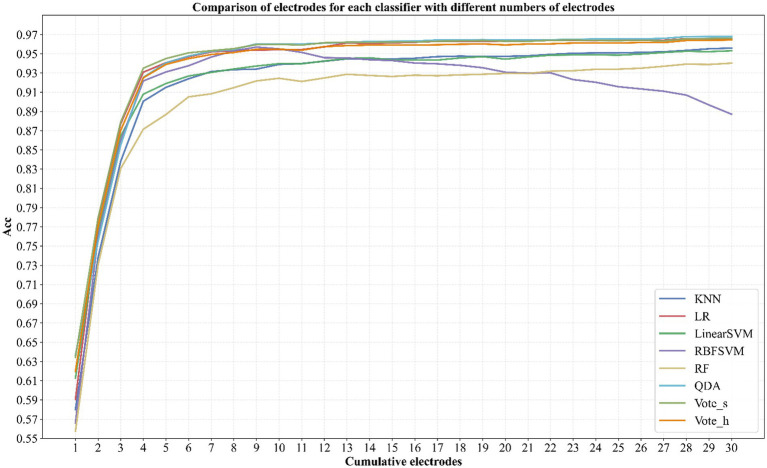
The average accuracy varies with the increasing of the number of subjects when using different classifier.

To systematically assess the effect of electrode count, we performed ten independent experiments for each cumulative electrode configuration, with N incrementally increased from 1 to 30, and calculated the mean accuracy. Figure 5 reveals a triphasic growth pattern in mean identification accuracy: a steep increase (1–4 electrodes), marginal gains (4–9 electrodes), and asymptotic stability (>9 electrodes) (*Δ* accuracy <0.5%, *p* > 0.05 for all classifiers except RBFSVM). The QDA classifier exemplifies this trend, achieving peak stability (96.0%) at 9 electrodes—only 0.8% below its 29-electrode maximum (96.8%). Subsequent electrode additions yielded no statistically significant improvement (*p* > 0.05), reinforcing that excessive electrodes increase hardware complexity and subject discomfort without meaningful gains. Thus, we propose a 9-electrode/QDA configuration as the optimal trade-off, achieving 96.0% accuracy while minimizing resource burden—a critical consideration for practical biometric systems.

In addition to the classifiers employed, the features extracted from diverse electrodes play a crucial role. Since EEG signals recorded from various electrodes reflect the functions and characteristics of distinct brain regions, they underpin the feasibility of EEG-based identification. Consequently, the unique characteristics of brain regions among individuals lead to variations in identification performance across different electrodes. As illustrated in Figure 5, statistical analysis of cumulative channel contributions reveals distinct inter-electrode variability in subject discriminability. Electrode TP8 exhibits greater universality, enabling the distinction of most subjects. Conversely, electrode Oz demonstrates greater homogeneity, with less pronounced individual characteristics, rendering it challenging to differentiate among subjects when used in isolation. This suggests that each subject may possess their own optimal electrode for achieving optimal identification performance. Given that normality and homogeneity of variance assumptions were violated, nonparametric, Wilcoxon signed-rank tests were applied to compare performance across eight classifiers and multiple electrode configurations (Table 2). Results revealed significant performance stratification: both QDA and Vote_s outperformed the other classifiers (*p* < 0.001, FDR-corrected). Although no statistically significant difference was found between QDA and Vote_s (*W* = 159.5, *p* = 0.144; $r_{\mathrm{rb}}\;$ = 0.314), the latter entails substantially higher computational cost due to its ensemble structure. Thus, for the 9-electrode configuration, QDA is recommended as it achieves comparable accuracy with considerably lower computational overhead—an essential balance for practical, deployable EEG biometric systems.

**Table 2:** Results of wilcoxon signed-rank tests for 8 classifiers.

Classifier	W	*p*	$r_{\mathrm{rb}}$	Median_diff
QDA, KNN	0.000	<0.001	1.000	0.017
QDA, LR	82.000	0.002	0.647	0.001
QDA, LinearSVM	3.000	<0.001	0.987	0.018
QDA, RBFSVM	9.000	<0.001	0.961	0.023
QDA, RF	0.000	<0.001	1.000	0.035
QDA, Vote_s	159.000	0.144	0.314	0.001
QDA, Vote_h	57.000	0.004	0.755	0.004
Vote_s, KNN	0.000	<0.001	1.000	0.017
Vote_s, LR	61.000	0.001	0.738	0.001
Vote_s, LinearSVM	0.000	<0.001	1.000	0.017
Vote_s, RF	0.000	<0.001	1.000	0.035
Vote_s, Vote_h	0.000	<0.001	1.000	0.036
KNN, LinearSVM	215.000	0.719	0.075	0.001
KNN, RBFSVM	163.000	0.164	0.299	0.006
KNN, RF	0.000	<0.001	1.000	0.018
KNN, LR	0.000	<0.001	−1.000	−0.015
KNN, Vote_h	0.000	<0.001	−1.000	−0.014
LR, LinearSVM	29.000	<0.001	0.875	0.016
LR, RBFSVM	6.000	<0.001	0.974	0.023
LR, RF	0.000	<0.001	1.000	0.033
LR, Vote_h	37.000	<0.001	0.841	0.003
LinearSVM, RBFSVM	131.000	0.043	0.437	0.005
LinearSVM, RF	0.000	<0.001	1.000	0.017
LinearSVM, Vote_h	0.000	<0.001	−1.000	−0.013
RBFSVM, RF	145.000	0.082	0.376	0.013
RBFSVM, Vote_s	0.000	<0.001	−1.000	−0.023
RBFSVM, Vote_h	7.000	<0.001	−0.970	−0.019
RF, Vote_h	0.000	<0.001	−1.000	−0.032

**Figure 6:**
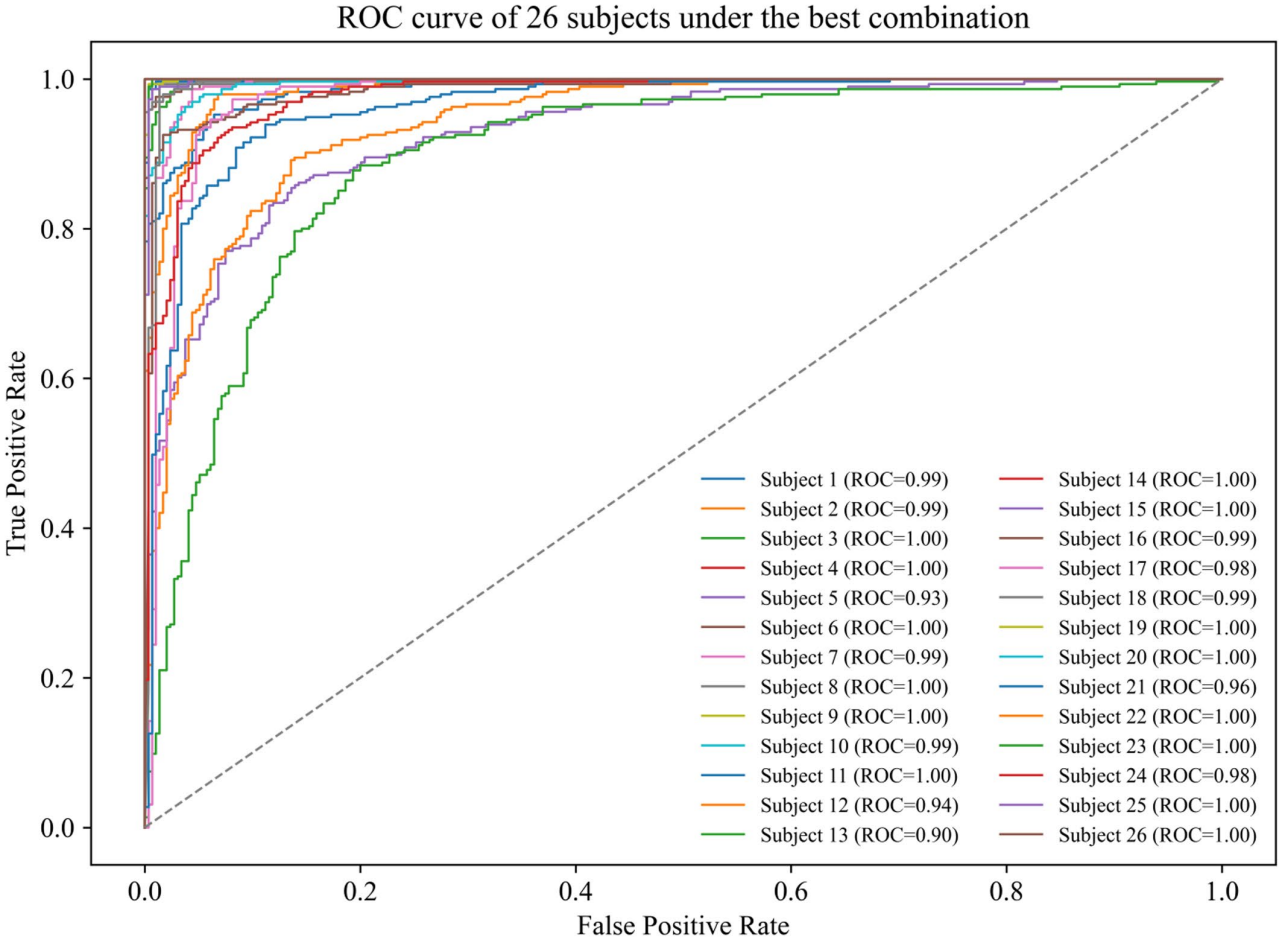
ROC curves demonstrating high-performance person authentication using optimized EEG configuration (9 electrodes, 13 entropy features, QDA).

To validate the optimal configuration—nine electrodes, thirteen entropy-based features, and a QDA classifier—we generated receiver operating characteristic (ROC) curves. Figure 6 shows for all 26 subjects, revealing outstanding classification performance. Notably, several subjects achieved a perfect AUC of 1.00, indicating flawless separability, while the majority attained AUC values exceeding 0.95, demonstrating high robustness.

**Figure 7:**
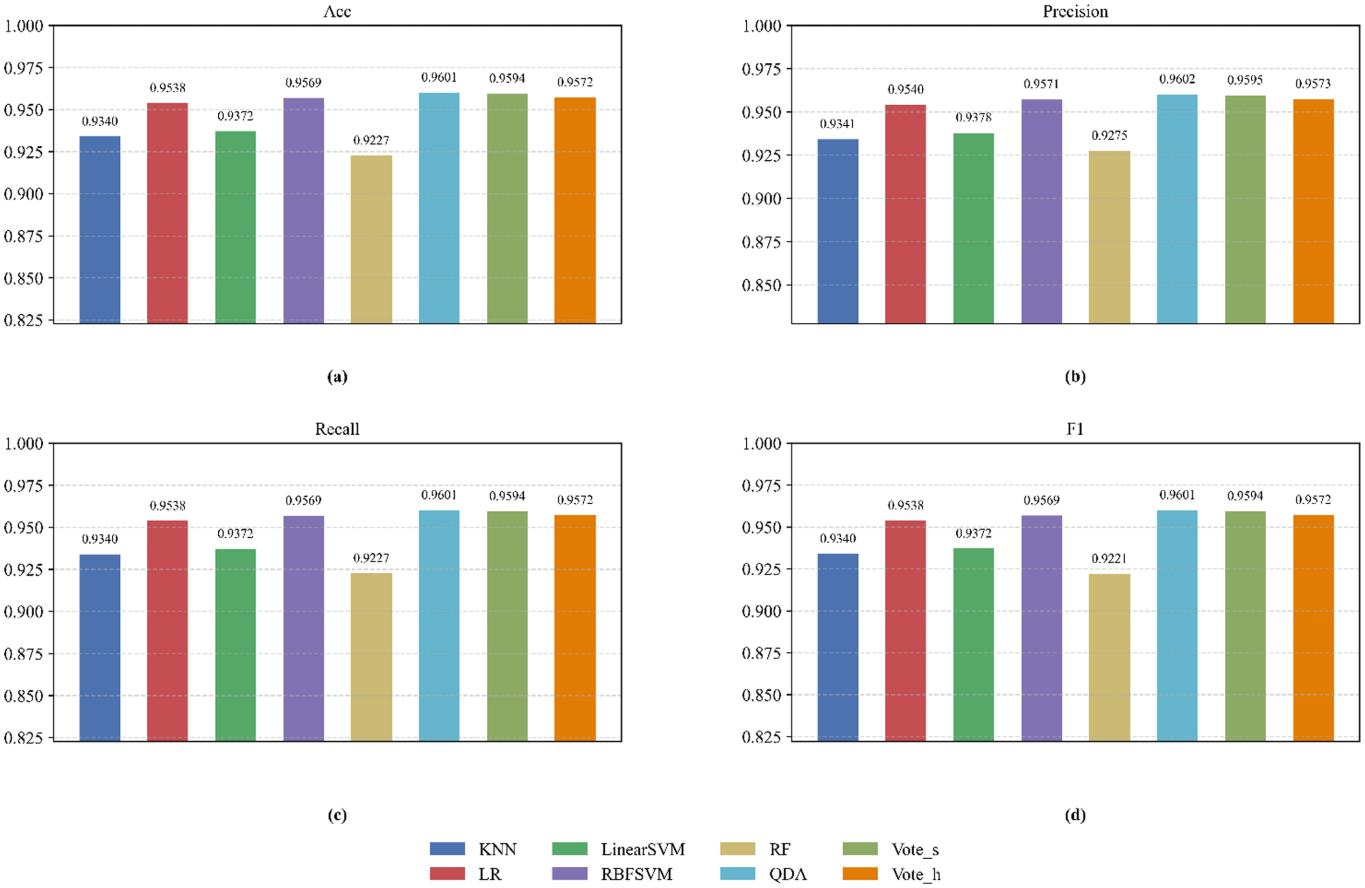
Classifier Performance Comparison: **(a)** Mean Accuracy, **(b)** Precision, **(c)** Recall, and **(d)** F1-Score for 13 entropy features from nine channel sets.

We quantified the performance of eight classifiers under optimal channel-feature combinations by computing mean accuracy, precision, recall, F1-score, and confusion matrices. As shown in Figure 7, QDA consistently achieved superior overall accuracy, significantly outperforming all counterparts, Ensemble methods (Vote_s and Vote_h) ranked second, while RBFSVM, Linear SVM, KNN, and LR demonstrated intermediate efficacy. In contrast, RF exhibited suboptimal performance across all metrics, aligning with its known limitations in high-dimensional EEG data. Table 3 further details the discriminative capability through confusion matrices. QDA exhibited the highest diagonal accuracy (reflecting correct class assignments) and optimally balanced precision-recall tradeoffs for both positive and negative classes. Specifically, it minimized misclassification rates (e.g., 2.4% inter-class confusion) while maintaining 96% class-wise precision and recall – a critical advantage for person identification robustness.

**Table 3:** Confusion matrix of eight model.

Classifier	Actual value/Predicted value	Negative	Positive
KNN	Negative	6,131	359
	Positive	347	6,143
LR	Negative	6,222	268
	Positive	330	6,160
LinearSVM	Negative	6,093	397
	Positive	287	6,203
RBFSVM	Negative	6,176	314
	Positive	244	6,246
RF	Negative	6,243	247
	Positive	625	5,865
QDA	Negative	6,233	257
	Positive	259	6,231
Vote_s	Negative	6,231	259
	Positive	266	6,224
Vote_h	Negative	6,234	256
	Positive	298	6,192

## 4 Discussion

Recently, several research groups have tackled the problem of utilizing EEG signals for person authentication (Boubakeur et al., 2017; Maiorana et al., 2015). Their approaches can be broadly categorized into three types:(1) Rest State EEG: Rocca et al. extracted biometric traits for person authentication using resting EEG signals, leveraging different recording sessions separated in time to demonstrate the repeatability of EEG features. By employing autoregressive statistical modeling and a linear classifier, a high degree of accuracy was achieved (La Rocca et al., 2013). (2) Event-Stimulated EEG: Yang et al. (2011) employed an event-related potential, specifically P300, to study person authentication using a learning vector quantization neural network, achieving a correct authentication rate of 92.1%. (3) Mental Imagery EEG: Nieves and Manian (2016) proposed an automatic person authentication system based on EEG signals derived from simple motor imagery movements, utilizing a minimum number of channels. A comprehensive review of EEG-based person authentication methods can also be found in Fraschini et al. (2018).

When compared to existing EEG-based authentication methods, the proposed method for person authentication exhibits improved performance with a more robust detector. Unlike many previous works that rely on event-related potentials (e.g.,Mu et al., 2017; Yeom et al., 2013) or task-induced mental imagery (Rodrigues et al., 2016), our approach utilizes resting-state EEG, which offers greater practicality for real-world applications by eliminating the need for active user participation or stimulus presentation equipment. Moreover, while some studies such as Maiorana et al. (2016) also employed resting-state EEG, they utilized substantially more electrodes (19 channels) compared to our configuration (9 channels), which increases setup complexity and subject discomfort. Our electrode reduction strategy, informed by systematic channel selection, demonstrates that comparable—in fact superior—accuracy can be achieved with fewer electrodes, enhancing usability without compromising performance.

Another critical differentiation lies in our feature extraction framework. While earlier studies predominantly utilized temporal, spectral, or wavelet-based features, we introduced a comprehensive entropy-based feature set that captures the nonlinear dynamics and complexity of resting-state EEG signals. Specifically, our findings indicate that SpEn outperforms other entropy measures, suggesting that frequency domain unpredictability provides particularly discriminative information for identity authentication. This represents a significant departure from conventional power-based or ERP-component features commonly used in prior literature.

Classifier selection further distinguishes our work. Whereas several previous studies employed Support Vector Machines (Mu et al., 2017; Yeom et al., 2013), k-NN (Maiorana et al., 2016), or neural networks (Yang et al., 2011), we implemented Quadratic Discriminant Analysis (QDA), which proved highly effective in modeling the distribution of entropy features. The superior performance of QDA (96.1% accuracy) compared to other classifiers in our study—and relative to those reported in prior works—may be attributed to its ability to handle non-linear class boundaries and different covariance structures across subjects, which is especially relevant given the high intersubject variability in EEG signals.

Furthermore, a comparison of related classification performances from previous studies is presented in Table 4. When compared to existing EEG-based identification/authentication methods, the proposed method for person authentication exhibits improved performance with a more robust detector. Given the reduced need for electrodes and the fusion of multiple classifier advantages, the proposed method is more convenient and effective.

**Table 4:** Performance comparison of the previous works.

Author	Classifier	EEG paradigm	Number of electrodes	Accuracy (%)
Maiorana et al. (2016)	Nearest-neighbor	Resting-state	19	87.9
Mu et al. (2017)	SVM	Event-Related Potential	22	90.7
Rodrigues et al. (2016)	Optimum-path Forest classifier	Motor Imagery	30	87.0
Yeom et al. (2013)	SVM	Event-Related Potential	5	86.3
This paper	QDA	Resting-state	9	96.1

The experimental results can be summarized as follows: (1) Utilizing a reduced number of electrodes for person authentication is feasible and practical. Specifically, the highest accuracy of 96.1% was achieved using nine electrodes and a QDA classifier, making it suitable, convenient, and comfortable for daily applications. (2) The significance of different electrodes in person authentication varies considerably. The electrode TP8 exhibited the best distinguishability for person authentication, with most subjects being distinguishable using this electrode. Conversely, the electrode Oz demonstrated the weakest distinguishability. (3) Among 13 entropy feature sets, SpEn exhibited superior person identification performance, whereas SaEn demonstrated significantly lower efficacy with marked differences from all other entropy measures. (4) Variations exist among classifiers for person authentication. The performance ranking of the eight classifiers was QDA ≈ vote_s > vote_h > radial basis function support vector machine (RBFSVM) > logistic regression (LR) > linear support vector machine (Linear SVM) > k-nearest neighbors (KNN) > random forest (RF).

However, this study has some limitations: (1) The sample size was relatively small, with only 26 subjects and 300 epochs per subject studied. For future research, we plan to recruit a substantial number of volunteers to further validate the performance. (2) Some classifier parameters were not optimized. As this work focused on evaluating the feasibility of the proposed method, finding the optimal classifier configuration conditions was beyond the scope of this paper. Future research will explore further performance improvements through parameter tuning. (3) The experiment was conducted in a controlled environment and has not been tested in outdoor natural settings, where various noise and uncertainty factors may affect authentication accuracy. In our future work, it will be meaningful to conduct further research in two areas: (i) validating the robust performance of person authentication with a larger pool of participants and (ii) exploring the actual application of an EEG-based person authentication system. For robust performance, it may be helpful to measure uncertainty in fusing different features and classifiers at an appropriate level in a given system, as well as to capture the sensitivity of the obtained results. For practical applications, although we performed an offline analysis on EEG datasets recorded from online experiments in this study, further research in a real-time online experimental environment is needed to confirm the findings. A real-time person authentication system using wireless EEG devices such as smartphones, tablets, and cloud servers could have widespread future applications. Therefore, a mobile person authentication system needs to be developed for online real-time applications. A global sensitivity and uncertainty analysis for the person authentication system will be beneficial in validating the robustness of the authentication results in the future. Considering convenience in practical applications, different priority channel regions and a reduced number of electrodes can be used for real-time person identity detection and authentication, which could benefit human authentication methods such as Brain Print or EEG passwords.

## 5 Conclusion

In this study, we propose an approach for a resting EEG-based system that utilizes thirteen entropy feature sets, six classifier types, and select electrodes to enhance the efficiency of person identity detection. The research findings demonstrate that the choice of feature sets and classifiers significantly impacts person authentication performance. Experimental results reveal that the proposed method achieves a commendable accuracy of 96.1% and offers a more robust detector. LOOCV suggests substantial potential applicability for person authentication using this approach. A resting EEG-based person authentication system promises valuable insights into biometric characteristics across relevant fields and serves as a significant supplement to existing methods.

## Data Availability

The original contributions presented in the study are included in the article/supplementary material, further inquiries can be directed to the corresponding author.
